# P-1053. Impact of Chlorhexidine Gluconate (CHG) Bathing on Bloodstream Infection Rates in Hematology-Oncology and Hematopoietic Stem Cell Transplant (HO/HSCT) Units

**DOI:** 10.1093/ofid/ofaf695.1248

**Published:** 2026-01-11

**Authors:** Yoona Rhee, Jae Jung, Michael Schoeny, Erik R Dubberke, Scott Fridkin, Erin Gettler, Surbhi Leekha, David K Warren, Matthew J Ziegler, Alexandra Seguin, Laura Rusie, Mary K Hayden, Michael Y Lin

**Affiliations:** Rush University Medical Center, Chicago, IL; Rush University Medical Center, Chicago, IL; Rush University College of Nursing, Chicago, Illinois; Washington University School of Medicine, Saint Louis, Missouri; emory university, Atlanta, Georgia; Duke University Hospital, Durham, NC; University of Maryland School of Medicine, Baltimore, Maryland; University of Nebraska Medical Center, Omaha, Nebraska; University of Pennsylvania, Philadelphia, Pennsylvania; Rush University Medical Center, Chicago, IL; Rush University Medical Center, Chicago, IL; Rush University Medical Center, Chicago, IL; Rush University Medical Center, Chicago, IL

## Abstract

**Background:**

Patients with hematologic malignancies are at high risk for healthcare-associated infections due to factors such as central lines and chemotherapy-associated mucosal barrier injury. In the intensive care unit, routine CHG bathing of patients reduces the risk of central line-associated bloodstream infections (CLABSIs); however, the impact of CHG in HO/HSCT units is less known.

**Figure 1.**
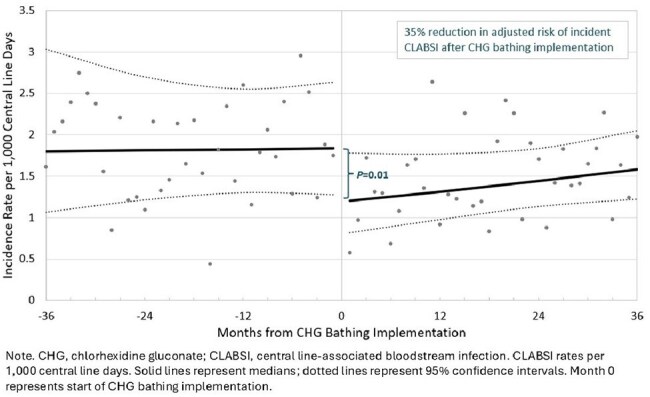
Modeled Central Line-associated Bloodstream Infection Rates Before and After Routine Chlorhexidine Gluconate Bathing Implementation in Hematology-Oncology/Hematopoietic Stem Cell Transplant Units from Six Hospitals

**Table 1.**
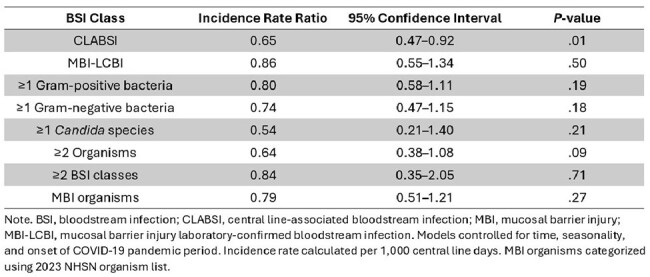
Modeled Incidence Rate Ratios of Bloodstream Infection Classes After Routine Chlorhexidine Gluconate Bathing Implementation in Hematology-Oncology/Hematopoietic Stem Cell Transplant Units from Six Hospitals

**Methods:**

This is a retrospective multicenter study of 6 hospitals. Each hospital obtained unit-attributable CLABSI and mucosal barrier injury laboratory-confirmed bloodstream infection (MBI-LCBI) events that were reported to the CDC National Healthcare Safety Network (NHSN) database, for 3 years before and after implementation of routine daily CHG bathing. BSIs that occurred during the years prior to the NHSN MBI-LCBI classification were adjudicated as either CLABSI or MBI-LCBI through chart review using NHSN rules and categorized by BSI subclasses. Monthly rates were calculated using line days. We used negative binomial regression to model the impact of CHG bathing on CLABSI and MBI-LCBI rates.

**Results:**

We compiled data from 11 HO/HSCT units with a median of 1.5 units per hospital. Bed capacity ranged from 16 to 48 beds/unit. A total of 775 CLABSI and 1053 MBI-LCBI events were identified between 2010 – 2022; the timing of CHG bathing implementation differed for each hospital. After implementation of routine CHG bathing, unadjusted CLABSI rates decreased from 1.85 to 1.48/1,000 central line days (*P*=.07); unadjusted MBI-LCBI rates increased from 1.95 to 2.51/1,000 central line days (*P*=.04). After controlling for time, seasonality, and onset of the COVID-19 pandemic period, there was a 35% reduction in the adjusted risk of incident CLABSI (incidence rate ratio, IRR 0.65 (95% confidence interval: 0.47–0.92, *P*=.01) after implementation of routine CHG bathing (Figure). There was no significant difference in the IRRs for MBI-LCBI and the BSI subclasses (Table).

**Conclusion:**

In this multicenter observational study of hematology/oncology and stem cell transplant patients, implementation of CHG bathing was associated with a significantly lower CLABSI incidence.

**Disclosures:**

Erik R. Dubberke, MD, MSPH, AstraZeneca: Advisor/Consultant|AstraZeneca: Grant/Research Support|Merck: Grant/Research Support|Pfizer: Advisor/Consultant|Pfizer: Grant/Research Support|Theriva Biologics: Grant/Research Support|Vedanta: Advisor/Consultant|Vedanta: Grant/Research Support David K. Warren, MD, MPH, Pfizer, Inc: Stocks/Bonds (Public Company)

